# Microglia Modulate Neurodevelopment in Autism Spectrum Disorder and Schizophrenia

**DOI:** 10.3390/ijms242417297

**Published:** 2023-12-09

**Authors:** Guangxiang Fan, Jiamin Ma, Ruyi Ma, Mingjiao Suo, Yiwen Chen, Siming Zhang, Yan Zeng, Yushan Chen

**Affiliations:** Brain Science and Advanced Technology Institute, Hubei Province Key Laboratory of Occupational Hazard Identification and Control, School of Medicine, Wuhan University of Science and Technology, Wuhan 430065, China

**Keywords:** microglia, autism spectrum disorder (ASD), schizophrenia (SZ), neurodevelopment, synapse development

## Abstract

Neurodevelopmental disorders (NDDs) include various neurological disorders with high genetic heterogeneity, characterized by delayed or impaired cognition, communication, adaptive behavior, and psychomotor skills. These disorders result in significant morbidity for children, thus burdening families and healthcare/educational systems. However, there is a lack of early diagnosis and effective therapies. Therefore, a more connected approach is required to explore these disorders. Microglia, the primary phagocytic cells within the central nervous system, are crucial in regulating neuronal viability, influencing synaptic dynamics, and determining neurodevelopmental outcomes. Although the neurobiological basis of autism spectrum disorder (ASD) and schizophrenia (SZ) has attracted attention in recent decades, the role of microglia in ASD and SZ remains unclear and requires further discussion. In this review, the important and frequently multifaceted roles that microglia play during neurodevelopment are meticulously emphasized and potential microglial mechanisms that might be involved in conditions such as ASD and SZ are postulated. It is of utmost importance to acquire a comprehensive understanding of the complexities of the interplay between microglia and neurons to design effective, targeted therapeutic strategies to mitigate the effects of NDDs.

## 1. Introduction

Neurodevelopmental disorders (NDDs) are complex disorders that can present early during childhood or later during adolescence and are characterized by sensory, motor, and cognitive dysfunctions that persist throughout an individual’s life [1]. Approximately 15–20% of children under 18 years of age are diagnosed with a developmental disability in the United States [2]. The therapeutic landscape for NDDs, including autism spectrum disorder (ASD), schizophrenia (SZ), and attention deficit hyperactivity disorder (ADHD), remains constrained [3]. ASD, a prominent NDD, is distinguished by compromised social and communicative abilities accompanied by repetitive behaviors, with symptom onset typically around the age of 3 years [4]. The median prevalence of ASD stands at 1 in 100, with males being four times more likely to be diagnosed than females [5]. SZ, a prevalent psychiatric ailment, is characterized by positive psychotic manifestations such as delusional thought processes, hallucinations, and erratic behaviors. It also encompasses negative symptoms such as diminished motivation, emotional blunting, social reclusiveness, and enduring neurocognitive impairments [6]. Both ASD and SZ are multifaceted disorders with intersecting symptomatic and pathophysiological features, particularly those associated with the immune system [1]. Microglia, which are central to brain development and equilibrium, may underlie the etiology of NDDs when their intrinsic function deviates. Contemporary perspectives on microglia underscore their profound influence across neuronal lifespan, from genesis to apoptosis [7]. Notably, deviations in microglial characteristics have been documented in both experimental models and clinical observations of patients with ASD and SZ [8]. This review explores the interplay between microglia and the neurodevelopmental trajectory of ASD and SZ and elucidates the modulatory role of microglia in these disorders.

## 2. Microglia in Neurodevelopment: Origin and Physiological Roles

### 2.1. Ontogeny and Maturation of Microglia

**Figure 1:**
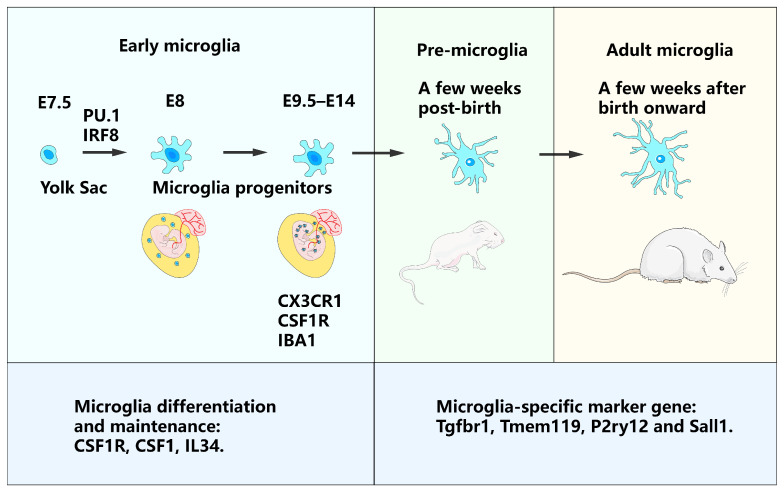
Microglia origin and development. Microglia are derived from the extra-embryonic yolk sac and differentiate into microglial progenitors by the transcription factors PU.1 and IRF8 around embryonic day 8 (E8). From E9.5 to E14, microglia begin to colonize the neural tube and express CX3CR1, CSF1R, and IBAl. Early microglia become pre-microglia and adult microglia following several stages of proliferation and differentiation since E14 is dependent on key factors such as CSF1R, CSF1, and IL34. Microglia-specific marker genes: Tgfbr1, Tmem119, P2ry12, and Sall1.

Microglia, which can be succinctly described as specialized macrophages that reside within the healthy brain parenchyma, constitute 5–20% of all glial cells in the central nervous system (CNS), as indicated by Kierdorf and Prinz [8]. Intriguingly, these cells originate from erythromyeloid precursors, which subsequently transform into microglial progenitors, specifically in the extraembryonic yolk sac around embryonic day 8 (E8). It is worth noting that this transformation and differentiation are remarkably contingent upon the nuanced interplay of the transcription factors spi-1/purine rich box-1 (PU.1) and interferon regulatory factor 8 (IRF8) during the intricate process of embryogenesis, as evidenced by Ginhoux et al. [9] (Figure 1).

From E9.5 to E14, these progenitors initiate migration from the yolk sac to the neural tube via blood circulation, which is marked by the upregulation of the fractalkine receptor, chemokine C-X3-C motif receptor 1 (CX3CR1), and the colony-stimulating factor 1 receptor (CSF1R) within the developing CNS, as illustrated by Ginhoux et al. and Kierdorf et al. [9,10]. Concurrently, ionized calcium-binding adaptor molecule 1 (IBA1), another vital microglial marker, plays a pivotal role in the successful integration of microglia into progressively maturing brains, as described by Kierdorf et al. [10]. Following several stages of proliferation and differentiation, mature microglia are firmly established within the parenchyma, where they manage to persist and renew autonomously and are remarkably unaffected by the peripheral immune system, as shown by Ajami et al. and Davoli-Ferreira et al. [11,12]. The differentiation and sustenance of microglia rely heavily on the mutual expression of CSF1R and its ligands, macrophage-stimulating factor 1 (CSF1), and interleukin (IL)34 [13,14]. Microglial development is promoted by CSF1 in the cerebellum and by IL34 in other brain regions [14]. Additionally, signaling via transforming growth factor β (TGF-β) has been shown to be necessary to drive the expression of microglia-specific marker genes, such as transforming growth factor beta receptor type I (Tgfbr1), transmembrane protein 119 (Tmem119), Purinergic Receptor P2Y12 (P2ry12), and spalt-like transcription factor 1 (Sall1), distinguishing microglia from bone marrow-derived macrophages [15,16] (Figure 1).

Microglial development follows a roughly stepwise differentiation and maturation program characterized by distinct transcriptional stages: early microglia (up to E14), pre-microglia (from E14 to several weeks post-birth), and adult microglia (several weeks post-birth and beyond) [17,18]. Each developmental stage is characterized by specific markers and regulatory elements [18]. Li et al. identified expression patterns related to embryonic, immature, proliferative, and homeostatic microglia [19]. Single-cell RNA sequencing (scRNA-seq) data revealed pronounced heterogeneity in early postnatal microglia compared with adult homeostatic microglia, indicating microglial heterogeneity during development [19]. Nevertheless, adult microglia display regional variations in morphology, density, membrane properties, and lysosomal content, suggesting the potential influence of microenvironmental factors on microglial region-specific phenotypes [20]. Environmental cues determine microglial phenotypes by affecting various tissue-specific enhancers that bind to the transcription factor PU.1 [21]. Microglia sense the extracellular milieu through “sensome” genes that are highly expressed in microglia, including Tmem119, Cx3cr1, triggering receptors expressed on myeloid cells 2 (Trem2), P2ry6, P2ry12, and P2ry13 [22]. However, the mechanisms and significance of heterogeneity and diversity in different periods of life and brain regions are topics of debate.

Following engraftment and proliferation, microglia drastically changed from an amoeboid-like morphology to a ramified phenotype [23]. This transformation appears to result from cell-autonomous and intrinsic developmental performance as well as a response to microenvironmental cues during development through neurons and other glial cells [20]. Reports have shown that stimulating bone marrow-derived macrophages to differentiate into microglia upon exposure to the adult brain environment [24]. However, cells originating from the yolk sac can undergo microglial differentiation and identity when exposed to the cellular microenvironment [25]. Moreover, neurons and their progenitors modulate microglial distribution and maturation because their ablation prevents microglial recruitment to specific regions [26]. These findings underscore the pivotal role of yolk sac-derived cells and microenvironmental cues during neurodevelopment in guiding microglial differentiation and growth.

Microglia exhibit two distinct phenotypes, M1 (classical activation state) and M2 (alternative activation state), which can switch from one type to another [27]. M1 microglia generate proinflammatory cytokines IL-1β, IL-6, and TNF-α, as well as neurotoxic elements such as reactive oxygen species (ROS). On the contrary, M2 microglia release anti-inflammatory mediators, such as IL-10, TGFβ, and neurotrophic factors, which induce neuroprotectivity [28,29]. M1 and M2 microglia are both related to the pathobiology of inflammatory and CNS disorders [30]. Various modulators regulate the switching of microglial phenotypes between M1 and M2, involving receptors, ion channels, transcription factors, and cytokines. These regulators are associated with signaling pathways such as nuclear factor kappa-B, Toll-like receptor, mitogen-activated protein kinases, and phosphatidylinositol 3-kinase/protein kinase B signaling pathways [30]. However, there is a series of intermediates phenotypes in microglia due to the varied microglia activation; thus, the M1/M2 paradigm is inappropriate to accurately describe microglia activation in vivo [31,32].

### 2.2. Physiological Functions of Microglia during Development

**Figure 2:**
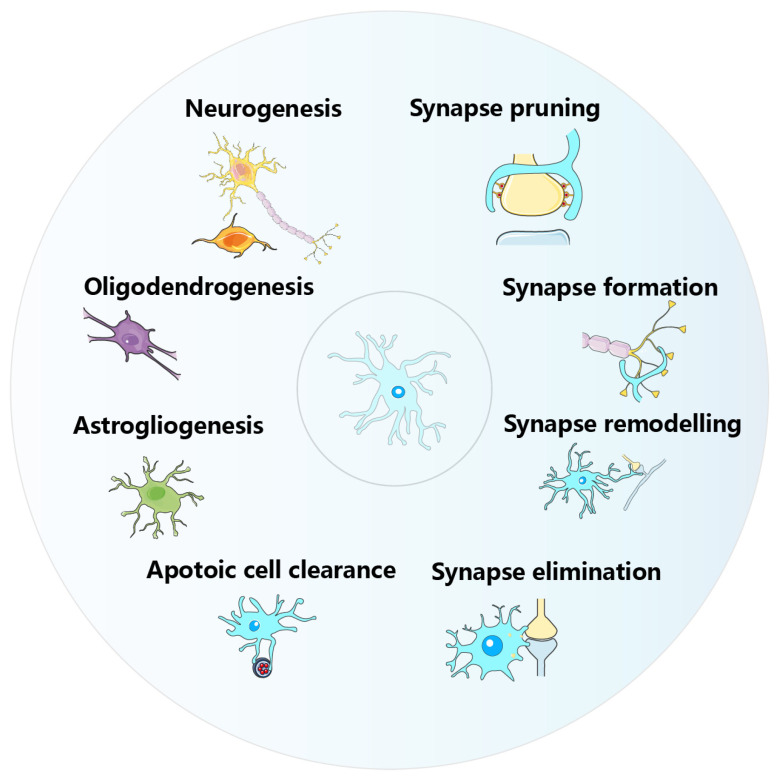
Main biological and physiological functions of microglia during development. Microglia modulate the proliferation and differentiation of neuronal, oligodendrocyte, and astrocyte precursor cells, as well as neuronal survival and apoptosis. Microglia also regulate synapse formation, remodeling, elimination, and pruning during development.

As resident macrophages of the CNS, microglia are pivotal in immunosurveillance and significantly influence brain development, maturation, and function during prenatal and early postnatal phases [7]. Accumulating evidence indicates that microglia contribute to key developmental events such as neurogenesis [33], astrogliogenesis [34], oligodendrogenesis [35,36], neuronal migration [33], neuronal cell death, and apoptotic cell clearance [7] as well as synapse elimination [37], formation [38], and pruning [39] (Figure 2).

During embryogenesis, microglia are recognized as pioneering glial cells that colonize the developing CNS. Their influence on neurogenesis is mediated by the secretion of neurotrophic or neurotoxic factors that coordinate the survival, differentiation, and apoptosis of early-stage neuronal progenitors [40,41]. For instance, microglial-derived superoxide ions trigger the apoptosis of Purkinje cells in the postnatal cerebellum [40]. Conversely, neuronal progenitors show enhanced survival and proliferation when they are co-cultured with microglia compared with solitary cultures [39]. Noteworthy observations include diminished neuronal progenitor counts in Csfr1-deleted mice [26] and cortical neurons that fail to survive in microglia lacking Cx3cr1 [42].

In addition to modulating neurogenesis, microglia also induce neuronal apoptosis during development. Programmed cell death (PCD) reduces excess neurons and facilitates proper neuronal connections [43,44]. Recent studies have revealed that microglia induce PCD through the secretion of various molecules. For instance, microglia expressing neurotrophic growth factor (NGF) induce PCD in the developing chick retina [45]. Similarly, microglia secrete tumor necrosis factor-alpha to promote neuronal cell death in the mouse spinal cord [46]. Brain macrophages trigger Purkinje neuron apoptosis in the postnatal mouse cerebellum via superoxide ion production [40].

Microglia critically influence synapse formation and maturation. Key factors secreted by microglia, such as brain-derived neurotrophic factor (BDNF) and interleukin 10 (IL10), induce the formation of both inhibitory and excitatory synapses in the hippocampus [47,48]. Additionally, Miyamoto et al. observed that abundant synaptogenesis occurs when microglia and spines contact the developing cortex, using real-time in vivo multiphoton imaging, indicating that microglial contacts regulate synapse formation during development [38]. Minocycline, a microglial inhibitor, curtails filopodium formation rates without altering dendritic-microglial contact frequency, underscoring the significance of the signaling interplay between microglia and the corresponding dendritic shaft for spine formation [49]. Ca^2+^ imaging revealed enhanced filopodium formation in dendrites near microglia, suggesting an increased Ca^2+^ concentration for spine formation due to microglial-dendritic interactions. Additionally, the removal of microglia leads to a marked decline in miniature excitatory postsynaptic current (mEPSC) frequency in sensory cortical pyramidal cells, emphasizing their indispensable role in spine development. The role of CX3CR1 in microglia during synaptic development in the brain has also been documented [50].

Microglia have consistently been shown to regulate synaptic elimination. During brain development, early excess synapses are gradually trimmed to normal numbers by microglia. The CX3CL1-CX3CR1 signaling axis was initially identified as instrumental in microglial synaptic pruning [39]. Pertinent findings revealed elevated spine density and excitatory postsynaptic current (EPSC) frequency in Cx3cr1 KO mice relative to wild-type controls. Schafer et al. later demonstrated microglial engulfment of synaptic elements in the lateral geniculate nucleus (LGN) via a complement-dependent mechanism [51]. Activity-dependent synaptic engulfment by microglia in the dorsal LGN, which is dependent on C3 or its receptor, CR3, has also been identified during eye segregation. Recently, several molecular players including TREM2, cluster of differentiation 47 (CD47), and phosphatidylserine (PS) have emerged as regulators of microglial synaptic pruning. For instance, Trem2-deficient mice present with elevated synaptic density and mEPSC frequencies in the hippocampal CA1 region, coupled with attenuated microglial synaptic phagocytosis [37]. Developmentally, the “do not eat me” signaling molecule CD47 and its receptor signal regulatory proteins α (SIRPα) in retinal ganglion cells (RGCs) show enhanced expression, with concomitant reductions in functional synapses and increased microglial-mediated synaptic phagocytosis, suggesting that CD47-SIRPα signaling modulates synapse elimination [52]. Furthermore, PS has been identified as a promoter of developmental synaptic elimination by microglia [53]. Notably, defective microglia-mediated synaptic pruning has been linked to enduring neural connectivity anomalies during development [37,54].

In conclusion, the findings uncovered by the abovementioned research shed light on the numerous and significant functions of microglia within the CNS. Their functions are extremely diverse as they are responsible for directing neurogenesis and protecting neuronal synapses. Microglia do not merely play a supporting role; rather, they play an active role in the formation of immature neuronal circuits, the regulation of neuronal viability, and the direction of connectivity throughout neurodevelopment. Microglia can navigate the intricate CNS environment with pinpoint accuracy because they are perfectly synchronized with the CNS environment and exist within a complex signaling network.

## 3. Microglial Implications in ASD

### 3.1. Aberrant Microglial Function in ASD

Converging data from neuropathological investigations of post-mortem brain specimens and positron emission tomography (PET) evaluations of living individuals with ASD have indicated the pervasive presence of dysregulated microglia in ASD [55]. Notably, in ASD-afflicted brains, microglia exhibit heightened densities, particularly in the cerebral and cerebellar cortices, coupled with morphological alterations, such as amplified cell bodies and retracted thickened processes [56,57]. Concurrently, these morphological deviations are accompanied by fluctuations in the microglial activation markers [56,58]. Given the association of ASD with cytokines, neurotoxins, neurotrophic agents, and other factors secreted from both quiescent and activated microglia, it is plausible that microglia modulate a spectrum of neuronal functions and shape synaptic connections via these mediators [39,54,59]. Core ASD symptoms are linked to anomalies in neurogenesis, aberrant synaptic pruning, and dysfunction during CNS maturation [60]. Considering the integral roles of microglia in orchestrating neurogenesis, immunomodulation, synaptic genesis, and pruning within the CNS, it is evident that perturbed microglial operations may be pivotal in ASD pathogenesis.

Within the context of ASD, microglial aberrations distinctly manifest as pathophysiological signatures, as corroborated by neuroimaging [55], neuropathological [56,61,62], and transcriptomic [63,64] data. The interpretation of PET studies employing ligands targeting the translocator protein (TSPO) in the brains of patients with ASD remains ambiguous, primarily because of the utilization of distinct radioligand generations. For instance, first-generation tracers signal ‘neuroinflammation’ across various cerebral regions, predominantly the cerebellum [55], whereas second-generation tracers suggest diminished reactivity in analogous regions [65,66]. Recent neuropathological analyses have highlighted a persistent inflammatory microglial profile, marked by reduced process numbers and lengths in the dorsolateral prefrontal cortex (DLPFC) [56], amplified miR155 expression in the amygdala [67], and elevated major histocompatibility complex II expression in the cerebellum and frontal cortex [62]. Transcriptomically, initial cortex-wide sequencing delineates ASD pathology converging toward neuronal and innate immune system dysregulation [63,68,69]. Successive single-cell studies have identified genes, predominantly in the upper layer projections, that correlate with the clinical severity of ASD in younger patients. Notably, genes such as forkhead box p2 (FOXP2), spleen tyrosine kinase (SYK), Fyn binding protein (FYB), and autism susceptibility gene 2 (AUTS2) are associated with microglial developmental modulation and activation in ASD [64]. Furthermore, microglial genes associated with immune signaling and phagocytosis, such as janus kinase 3 (JAK3) and interferon alpha and beta receptor subunit 2 (IFNAR2), demonstrate pronounced upregulation in primary sensory regions in ASD [70].

During developmentally delicate periods, aberrantly activated microglia release pro-inflammatory cytokines, including TNF-α, IL-6, IL-8, and interferon γ (IFN-γ). This can alter synaptic homeostasis and disrupt neural connectivity, contributing to neurodevelopmental dysfunction [71,72]. Neuropathological studies of ASD post-mortem tissues have shown evidence of increased microglial activation and neuroinflammation in multiple brain regions [56,73]. Brain transcriptome immune findings have documented enrichment of the immune module, such as M2 microglia markers and type I interferon in the ASD cortex [68]. The dysregulation of innate inflammatory responses and microglial activation have also been identified in ASD preclinical studies. Polyinosinic-polycytidylic acid [Poly(I:C)] or lipopolysaccharide (LPS) induced maternal immune activation (MIA) model exhibited increased inflammatory cytokine production and peripheral IL-1β and IL-6 throughout growth [74,75]. Although the exact mechanisms remain unclear, the activated microglia and proinflammatory cytokines play an essential role in neurodevelopment.

Although compelling evidence points toward alterations in microglial activity as a characteristic feature of the brain of a person with ASD, the initiators of these microglial responses remain unclear. This is despite the fact that a relationship exists between ASD and microglial activity. Additionally, it remains unclear whether microglial dysfunction is the direct cause of ASD or whether it merely exacerbates symptoms that are already present. As a result, murine models have become essential resources for elucidating these mechanisms and developing targeted interventions, both of which are discussed in greater depth in the following sections.

### 3.2. Mice Models Employed to Explore Microglial Effects to ASD

An important question arises as to whether microglial activation merely serves as a secondary outcome of atypical brain development or whether it actively instigates or exacerbates ASD. In the quest for answers, a wide variety of genetic and environmental rodent models have been used to clarify the role that microglia play in the pathogenesis of ASD.

**Figure 3:**
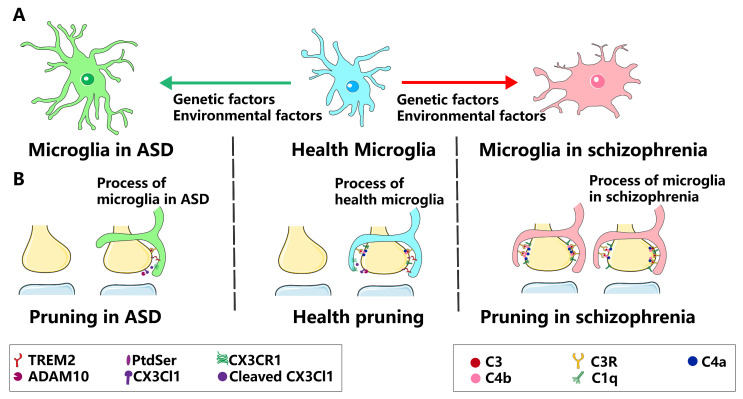
Microglial synaptic pruning in ASD and SZ. (**A**) Microglia exhibit abnormal synaptic pruning in ASD and SZ due to genetic and environmental factors. (**B**) Healthy microglia exhibit healthy pruning dependent on key regulators, including TREM2, C3, C3R, and PtdSer, at normal levels. In ASD, Microglia exhibit insufficient synaptic pruning because of the reduction in TREM2 and CX3CR1 protein levels in ASD individuals and animals. In schizophrenia, microglia-mediated hyperramified synapse pruning is mediated by overexpression of complementary proteins.

In rodent models, the genetic modulation of microglial activity can induce pronounced alterations in CNS function, leading to behavioral anomalies that mirror ASD characteristics. For instance, recent murine investigations have revealed that the lack of TREM2 induces aberrant neuronal synapse remodeling, imbalances in excitatory/inhibitory neurotransmission, compromised neuronal connectivity, and behavioral manifestations akin to ASD [37]. Post-mortem brain analyses of both neurotypical individuals and patients with ASD corroborated these findings, indicating diminished TREM2 protein levels in individuals with ASD. Interestingly, the most significant reduction in TREM2 was observed in individuals with pronounced ASD symptoms, indicating an inverse relationship between TREM2 concentration and ASD symptom severity [37] (Figure 3).

Trem2-deficient mice, characterized by hampered microglia-mediated synapse elimination, display ASD-associated behaviors, including repetitive actions and diminished social engagement [37]. Additionally, a 2017 study by Kim et al. demonstrated that mice deficient in Atg7, an autophagy-associated gene specific to bone marrow-derived cells, including microglia, manifested behaviors characteristic of ASD [76]. In this Atg7-deficient model, there was an increase in spine density and synapse-associated proteins in the sensory cortex. Notably, these mice exhibited increased PSD95 puncta within microglial cells, suggesting that synapses engulfed by microglia remained undigested due to autophagic dysfunction, subsequently inhibiting further synaptic phagocytosis.

Microglial-mediated synaptic modulation is emphasized in rodent models that reflect symptomatic ASD variants such as Fragile X Syndrome (FXS) and Tuberous Sclerosis (TSC). These models highlight the central role that microglia play in ASD development. When there is an abnormally high number of codon repetitions, fragile X messenger ribonucleoprotein 1 (Fmr1), the gene responsible for FXS, is located on the X chromosome and unable to produce a standard FMR protein. This disrupts the normal brain development. Compared to their counterparts with the wild-type genotype, mice deficient in Fmrl exhibited an increase in both size and number of microglial cells. This morphological shift correlates with attenuated microglia-facilitated synaptic pruning [77,78]. Jawaid et al. posited that the increased spine density in Fmrl-deficient mice stems from disrupted synapse elimination during maturation with marked reductions in microglial PSD95 phagocytosis [79]. The depletion of Fmrl also induces synaptic functional plasticity anomalies, potentially curtailing activity-dependent synaptic elimination. Patients with Rett syndrome and mutations in methyl-CpG-binding protein 2 (MECP2) exhibit ASD-like behaviors. Microglial-specific MECP2 deletions in mice induce glutamate overproduction, which, in turn, distorts neuronal shape and hinders synaptogenesis [80]. TSC, which stems from Tsc1 or Tsc2 mutations, is another genetic disorder with pronounced ASD comorbidities. Tsc2-deficient mice exhibit social memory deficits attributed to aberrant mammalian targets of rapamycin-driven interferon signaling and microglial dysfunction, highlighting the prominence of microglia in the nexus between early immune responses and ASD-linked genetic aberrations [81]. The overexpression of the Fragile X Messenger Ribonucleoprotein (FMRP) target eIF4E in microglia augments spine density and engenders ASD-like behaviors, mirroring the phenotypes observed in global eukaryotic initiation factor 4E (eIF4E) overexpressing mice [77,82,83]. Moreover, the targeted disruption of the core autophagy gene Atg7 in LYZ2^+^ myeloid cells, which potentially encompasses microglia, disrupts synaptic pruning [76]. Recent studies have revealed that deficiency in glutaminase 1, an enzyme vital for brain glutamate synthesis, compromises microglial synapse pruning and culminates in ASD-like manifestations [84].

However, considering that both quiescent and activated microglia can release cytokines, neurotoxins, neurotrophic factors, and other soluble mediators implicated in ASD, it is conceivable that microglia deploy these agents to modulate a spectrum of neuronal functions and orchestrate synaptic connections [39,54,59].

Environmental determinants also influence microglial functionality, thereby affecting neurodevelopment, synaptic interconnectivity, and CNS immune reactions [85]. The MIA paradigm involves prenatal exposure to agents such as Poly(I:C) or LPS and mirrors the epidemiological findings connecting prenatal infections to neurodevelopmental disorders, including ASD [86]. Newborn microglia from MIA-exposed environments display a premature downregulation of early developmental genes, such as spi-1 proto-oncogene (Spil) and interferon regulatory factor 8 (Irf8), transitioning toward an adult microglial transcriptional profile [61,87]. In a pivotal study, Andoh et al. identified deficient developmental synapse elimination in the hippocampus of MIA-induced ASD mouse models [88]. Interestingly, decreased synaptic phagocytosis by microglia increases the synaptic density. Reduced synaptic phagocytosis by microglia was found to cause increased synaptic density. However, when spontaneous exercise was introduced after ASD development in adulthood, it promoted microglial synaptic phagocytosis. This normalized synaptic density, which helped alleviate ASD-related behaviors in turn. Because of the increased activity that occurs in certain granule cells of the dentate gyrus as a result of physical activity, it is plausible that activity-dependent synaptic competition may be the driving force behind microglial synaptic phagocytosis.

Overall, these findings lend credence to the theory that microglia play an important role in ASD pathogenesis by influencing neuroimmune interactions, synaptic remodeling, neuronal survival, and connectivity.

## 4. Microglia in SZ

### 4.1. Genes, Microglia, and the Complement System in SZ

Post-mortem studies have revealed reduced dendritic spine density in the cortices of patients with SZ, suggesting that synaptic deficits are a potential cause of SZ [89]. Synapses from patients with SZ exhibit heightened susceptibility to microglial phagocytosis compared to healthy controls. Complement system genes also show a pronounced association with SZ [90]. Notably, Sekar et al. identified an SZ risk-enhancing allele of C4 in the major histocompatibility complex (MHC) locus [91], prompting investigations into the link between the complement system and SZ. A study conducted in Sweden by Cooper et al. (2017) supported the link between C4 and SZ and suggested the potential for predicting the risk of this disorder through neonatal blood analysis [92]. Elevated levels of brain microglial markers (TSPO) correlate with increased C4 levels in living human brains [93]. Moreover, C4-deficient mice display suppressed synaptic pruning [91]. The overexpression of C4A correlates with augmented synaptic complement C3 deposition, likely amplifying microglial synaptic phagocytosis [94] (Figure 3). Elevated C4A-mediated synaptic elimination aligns with the observed SZ pathologies, with parallels noted for C4A overexpression [95]. Distinctively, cerebrospinal fluid (CSF) C4 activity surges only in males in their twenties, potentially explaining the male-skewed prevalence of SZ [96]. Elevated C4 levels have also been observed in CSF [97]. Post-mortem samples from the midbrain and prefrontal cortex (PFC) of patients with SZ also exhibited upregulated complement pathway genes, such as C1qA [98] and C4 [99].

Emerging research underscores the relevance of the complement system in SZ, highlighting the potential diagnostic and therapeutic power of monitoring and modulating microglial synaptic pruning within this framework.

### 4.2. Microglia Interact with Synapses in SZ

During typical cerebral maturation, dendritic spine densities in the cortex are markedly higher during childhood than in adulthood, with a two-to-three-fold difference [100]. Predominantly during adolescence, microglia facilitate significant synaptic spine elimination, allowing the spine and synaptic densities to align with adult levels. Overzealous synaptic pruning during adolescence may underlie SZ etiology. Microglia-like cells (iMG) cultured from SZ patient-derived monocytes exhibit heightened synaptosome phagocytosis compared to control-derived iMG [94]. Microglia from patients with SZ, when co-cultured with neurons derived from pluripotent stem cells, resulted in a decrease in neuronal spine density. These findings indicate the intrinsic hyperactivity of SZ brain microglia in the elimination of phagocytic spines, which may explain the decreased cortical synaptic density observed in SZ. However, few studies have focused on synaptic pruning during adolescence, resulting in ambiguous comparisons with the earlier developmental stages. Atypical developmental synaptic pruning may be a factor in ASD, and abnormal adolescent synaptic pruning may cause SZ. Disruptions in SZ-related γ-band oscillations within cortical activity, which reflect cortical synaptic activities, can cause problems in cognition, perception, and consciousness [101].

Microglial processes, which are known for their mobility, effectively interact with boutons and spines. In particular, phagocytic microglial processes engulf pre- and post-synaptic elements during postnatal weeks [79,91]. Intriguingly, excessive synaptic pruning in the SZ is implicated in both neuronal and microglial dysfunction. Although SZ and control patient-derived neuronal cultures exhibited comparable spine densities, SZ-associated C4 risk variants increased C3 deposition (Figure 3). Moreover, co-culturing SZ patient-derived neurons with the corresponding iMG reduced spine density relative to control-derived cultures, bolstering the theory of complement-mediated excessive synaptic pruning in the SZ [94]. These findings provide a deeper mechanistic analysis of the neuronal and microglial roles in SZ. Maternal perinatal infections, known as SZ risk amplifiers, purportedly reduce offspring brain synaptic density, potentially influencing the brain complement system and escalating SZ risk [102,103]. Drawing from research demonstrating how maternal infection diminishes synaptic density in the brain of offspring [104,105], it is evident that such infections can influence the brain complement system and potentially elevate the risk of SZ. Single nucleotide variants in ST8 α-N-acetyl-neuraminide α-2,8-sialyltransferase 2 (ST8SIA2), which encode polysialyltransferases, are correlated with SZ. ST8SIA2-deficient rodents exhibit disrupted mossy fiber development and compromised fear-conditioned memory [106].

Additionally, post-mortem analyses revealed diminished dendritic spines, especially in the layer III PFC pyramidal neurons. Dendritic pruning, a neurodevelopmental experience-dependent plasticity process, consolidates certain neural connections while discarding others. Exaggerated late adolescent and early adult dendritic spine pruning might herald the onset of SZ symptoms during these pivotal phases [18,62,107].

Speculations have arisen regarding whether microglial anomalies precede or result from SZ. Animal models show neurodevelopmental microglial abnormalities. However, the precise role of microglia in SZ remains unclear.

## 5. Conclusions

The primary objective of this review is to highlight the fundamental role of microglial interactions, particularly those involving synapses, in actively facilitating the formation of synapses and ensuring that network functionality operates at its optimum level. We put forth the hypothesis that any type of disruption, aberration, or functional anomaly occurring within these essential microglial interactions may play a pivotal, potentially determining role in the initial stages of a variety of neurodevelopmental disorders as well as their subsequent progression. Unquestionably, the burgeoning and rapidly expanding body of research exploring the nuanced interplay between microglia and neurodevelopment, especially within the specific contexts of ASD and SZ, holds significant promise for future exploration and applications in the field. This is because ASD and SZ are two conditions that are associated with microglial dysfunction. An investigation that is both more in-depth and nuanced into the specific functions of microglia-specific genes has the potential to unearth novel and hitherto unknown mechanisms that are essential for neurodevelopmental health.

## Data Availability

Not applicable.
